# Water Molecular Dynamics in the Porous Structures of Ultrafiltration/Nanofiltration Asymmetric Cellulose Acetate–Silica Membranes

**DOI:** 10.3390/membranes12111122

**Published:** 2022-11-09

**Authors:** João Cunha, Miguel P. da Silva, Maria J. Beira, Marta C. Corvo, Pedro L. Almeida, Pedro J. Sebastião, João L. Figueirinhas, Maria Norberta de Pinho

**Affiliations:** 1 Center of Physics and Engineering of Advanced Materials (CeFEMA), Laboratory for Physics of Materials and Emerging Technologies (LaPMET), Instituto Superior Técnico (IST), Universidade de Lisboa (ULisboa), Av. Rovisco Pais 1, 1049-001 Lisboa, Portugal; 2 Department of Physics (DF), Instituto Superior Técnico (IST), Universidade de Lisboa (ULisboa), Av. Rovisco Pais 1, 1049-001 Lisboa, Portugal; 3 Department of Chemical Engineering (DEQ), Instituto Superior Técnico (IST), Universidade de Lisboa (ULisboa), Av. Rovisco Pais 1, 1049-001 Lisboa, Portugal; 4 Centro de Investigação em Materiais (CENIMAT), Faculdade de Ciências e Tecnologia, Universidade Nova de Lisboa, Campus da Caparica, 2829-516 Caparica, Portugal; 5 Department of Physics, ISEL, R. Conselheiro Emídio Navarro 1, 1959-007 Lisboa, Portugal

**Keywords:** NMR, spectroscopy, diffusometry, relaxometry, cellulose acetate, asymmetric membranes, ultrafiltration, nanofiltration

## Abstract

This study presents the characterization of water dynamics in cellulose acetate–silica asymmetric membranes with very different pore structures that are associated with a wide range of selective transport properties of ultrafiltration (UF) and nanofiltration (NF). By combining ${}^{1}$H NMR spectroscopy, diffusometry and relaxometry and considering that the spin–lattice relaxation rate of the studied systems is mainly determined by translational diffusion, individual rotations and rotations mediated by translational displacements, it was possible to assess the influence of the porous matrix’s confinement on the degree of water ordering and dynamics and to correlate this with UF/NF permeation characteristics. In fact, the less permeable membranes, CA/SiO${}_{2}$-22, characterized by smaller pores induce significant orientational order to the water molecules close to/interacting with the membrane matrix’s interface. Conversely, the model fitting analysis of the relaxometry results obtained for the more permeable sets of membranes, CA/SiO${}_{2}$-30 and CA/SiO${}_{2}$-34, did not evidence surface-induced orientational order, which might be explained by the reduced surface-to-volume ratio of the pores and consequent loss of sensitivity to the signal of surface-bound water. Comparing the findings with those of previous studies, it is clear that the fraction of more confined water molecules in the CA/SiO${}_{2}$-22-G20, CA/SiO${}_{2}$-30-G20 and CA/SiO${}_{2}$-34-G20 membranes of 0.83, 0.24 and 0.35, respectively, is in agreement with the obtained diffusion coefficients as well as with the pore sizes and hydraulic permeabilities of 3.5, 38 and 81 kg h${}^{-1}$ m${}^{-2}$ bar${}^{-1}$, respectively, reported in the literature. It was also possible to conclude that the post-treatment of the membranes with Triton X-100 surfactants produced no significant structural changes but increased the hydrophobic character of the surface, leading to higher diffusion coefficients, especially for systems associated with average smaller pore dimensions. Altogether, these findings evidence the potential of combining complementary NMR techniques to indirectly study hydrated asymmetric porous media, assess the influence of drying post-treatments on hybrid CA/SiO${}_{2}$ membrane’ surface characteristics and discriminate between ultra- and nano-filtration membrane systems.

## 1. Introduction

It is established that the structure and dynamics properties of pore-confined molecules are greatly affected by the morphology of porous media [1,2,3]. In membrane separation, the state of water within a membrane’s three-dimensional porous network plays a role in elucidating the mechanisms of its selective mass transfer task. Concertedly, the separation performance of a membrane can be gauged by the interplay of factors such as the pore size, electrical charge, hydrophilic/hydrophobic characteristics of the membrane polymeric or hybrid matrix and the solutes [4,5]. Therefore, the membranes’ porous structure and the state of water within its porous matrix are crucial to understanding the mechanisms of membrane selective transport.

The determination of the accurate morphological features of porous media still represents a challenge as many properties depend not only on the void size’s distribution but also on their connectivity and liquid–surface interactions [6]. Although there is a vast amount of scientific literature focused on microscopic and spectroscopic characterisation for elucidating the mechanisms of membrane selective transport in the active layer structures of integrally skinned cellulose acetate (CA) or cellulose esters membranes [4,7,8,9,10,11,12], this subject is more complex in the study of hybrid CA and silica, CA/SiO_2_, asymmetric membranes constituting the system of this work [13,14]. Previous studies by de Pinho et al. [15,16] on the characterisation of the water order and dynamics in asymmetric CA/SiO_2_ hybrid membranes, covering a wide range of ultrafiltration (UF) and nanofiltration (NF) permeation properties, pointed to an essential indication that Nuclear Magnetic Resonance (NMR) relaxometry observables, which are strongly dependent on water–surface interactions due to confinement, can be reliably correlated with the membranes’ asymmetric porous structures and selective permeation performance.

Nuclear Magnetic Resonance (NMR) relaxometry is a widely used experimental technique that enables the study of a large variety of chemical compounds, such as liquid crystals, polymers, ionic liquids and complex food systems, just to name a few [17,18,19,20]. The ^1^H NMR longitudinal relaxation rate dispersion (R${}_{1}$ in the function of the ${}^{1}$H Larmor frequency) is sensitive to molecular motions occurring at timescales ranging from milli- to picoseconds and from slower collective motions in liquid crystalline phases to fast molecular rotations. ${}^{1}$H NMR relaxometry is especially sensitive to the existence of some degree of confinement, enabling an indirect study of a confining matrix by introducing a well-known liquid, usually water, into its structure. Relaxation-inducing interactions of the probing liquid with the surrounding surfaces, often referred to as rotations mediated by translational displacements, enables the characterization of a given matrix in terms of the effective mean square displacement of the liquid molecules confined in the porous system as well as the degree of order induced by these interactions [21,22,23,24].

In the present work, the main objective is to probe the water molecular dynamics within the porous structure of asymmetric CA/SiO_2_ hybrid membranes over a wide range of UF and NF permeation properties by ^1^H NMR relaxometry as a means to assess the effect of the drying post-treatments on the membranes’ asymmetric structure modification.

## 2. Experimental Section

### 2.1. Membrane Preparation and Characterization

A series of flat asymmetric CA/SiO_2_ hybrid membranes were made in a laboratory by coupling the wet phase inversion [25] with sol–gel techniques [26]. The synthesis methodology is described by de Pinho et al. [13]. Membranes were made from casting solutions containing 16.4 wt.% cellulose acetate (CA) polymer (≈30,000 average molecular weight), supplied by Sigma-Aldrich (Steinheim, Germany), a SiO_2_ content equal to 5 wt.%, and three different solvent system ratios of formamide (enhancing pore-forming agent) and acetone. The acid catalysed hydrolysis of the SiO_2_ alkoxide sol–gel precursor was promoted in situ by adding deionised water, tetraethyl orthosilicate (TEOS), supplied by Sigma-Aldrich (Steinheim, Germany), and nitric acid to the polymer casting solution. All chemicals were of reagent grade and 65% nitric acid was of technical grade. Membrane films were cast with the aid of a 250 μm calibrated doctor blade, followed by evaporation for 30 s before coagulation in an ice-cold deionised water bath. Table 1 shows the membranes’ casting solution compositions and film-casting conditions used in the preparation of three membranes with distinct UF porous structures, labelled as CA/SiO_2_-22, CA/SiO_2_-30 and CA/SiO_2_-34. In these membrane labels, the second field is represented by numbers 22, 30 and 34, which correspond to the formamide contents of 21.3%, 29% and 32.9% (wt.%), respectively, in the casting solutions.

Following preparation, the asymmetric CA/SiO_2_ hybrid membranes were conditioned in surfactant mixtures by a procedure adapted from Vos et al. [27]. This treatment was carried out using aqueous solutions of non-ionic surface-active agents composed of glycerol, supplied by PanReac (Darmstadt, Germany), and/or triton X-100, supplied by VWR (Briare, France). In that regard, membrane films were immersed for 15 min in one of the following solutions: (a) an aqueous solution of glycerol 20 vol.% (G20) or (b) an aqueous solution of triton X-100 4 vol.% and glycerol 20 vol.% (GT). All chemicals used in the treatments were of reagent grade and the conductivity of the deionised water was lower than 10 μS cm${}^{-1}$. For NMR sample preparation, to access the water behavior within the membranes’ porous matrices, the membrane films were immersed in deionised water for 48 h. Excess surface water was gently removed before enclosing a roll of hydrated membrane film in a sealed 5 mm outer diameter NMR tube. The membranes are identified throughout this work by a three-field code: the first code refers to the membrane hybrid matrix (CA/SiO_2_), followed by a second field relative to the formamide content (in wt.%) in the casting solutions (of 22, 30 and 34), and the third corresponds to the drying membrane post-treatment of G20 or GT.

The membranes were characterised in terms of pure water hydraulic permeability (L${}_{p}$) and a molecular weight cut-off (MWCO) referring to the molecular weight of the solute that is 95 % retained by the membrane. Details on the characterisation of the membranes studied are described in da Silva et al. [15].

### 2.2. Methods

**${}^{1}$H NMR Spectroscopy**: The series of spectra obtained from the high resolution ${}^{1}$H NMR relaxometry experiments performed at 7T was analyzed in order to extract the number of Lorentzian components and their respective longitudinal relaxation rates and signal contribution.

**${}^{1}$H NMR Diffusometry**: At controlled temperatures and using a probe head with field gradient coils (Bruker Diff 30, Billerica, MA, USA) and a Bruker 7T superconductor connected to a Bruker Avance III NMR console, it was possible to measure the self-diffusion coefficient, *D*, of water molecules entrapped in the membrane matrix. The applied Pulsed Gradient Stimulated Echo (PGSE) sequence produces an attenuation of the signal intensity for increasing magnetic field gradient strengths, expressed by Equation (1):

(1) $$I=I_{0}exp\left\{-\gamma_{{}^{1}H}^{2}g^{2}D\delta^{3}\left(\frac{\Delta }{\delta }-\frac{1}{3}\right)\right\},$$

where $\gamma_{{}^{1}H}$ is the proton gyromagnetic ratio, *g* is the gradient strength, δ is the length of the gradient pulses and Δ is the delay between pulsed gradients. Expression (1) does not take into account that water molecules are confined, which means that the obtained diffusion coefficients can be viewed as having apparent values with an order of magnitude that is well-estimated. More exact estimations of the diffusion coefficients would require the development of robust models that take into account the experimental conditions, namely magnetic field gradient pulse durations, which, as far as the authors know, were not yet achieved. In the case of the studied systems, except for pure water, multi-exponential decays were observed, which lead to the addition of the corresponding number of components to Equation (1).

**${}^{1}$H NMR Relaxometry**: The longitudinal relaxation rate, $R_{1}$, was measured across a broad frequency range at controlled temperatures. For ${}^{1}$H Larmor frequencies ranging between 10 kHz and 9 MHz, the measurements were made using a home-developed Fast Field Cycling (FFC) relaxometer [28]. For the remaining frequencies, the conventional inversion recovery technique was applied using the Bruker Avance II console paired with a variable field iron-core Bruker BE-30 electromagnet (10–100 MHz) or with a Bruker Widebore 7T superconductor magnet for the measurements at 300 MHz.

## 3. Results and Discussion

### 3.1. Membrane Characterization

Table 2 shows the hydraulic permeability, L${}_{p}$, and molecular weight cut-off, MWCO, of the asymmetric CA/SiO${}_{2}$ hybrid membranes.

As it can be observed by looking at the hydraulic permeabilities previously obtained by de Pinho et al. [15] for the membrane systems studied in the present work, the CA/SiO${}_{2}$-30 and CA/SiO${}_{2}$-34 membranes present marked ultrafiltration characteristics, whereas the CA/SiO${}_{2}$-22 membrane has a hydraulic permeability tjhat is one order of magnitude lower, thus standing within the border between nano- and ultrafiltration.

### 3.2. ^1^H NMR Spectroscopy

**Figure 1:**
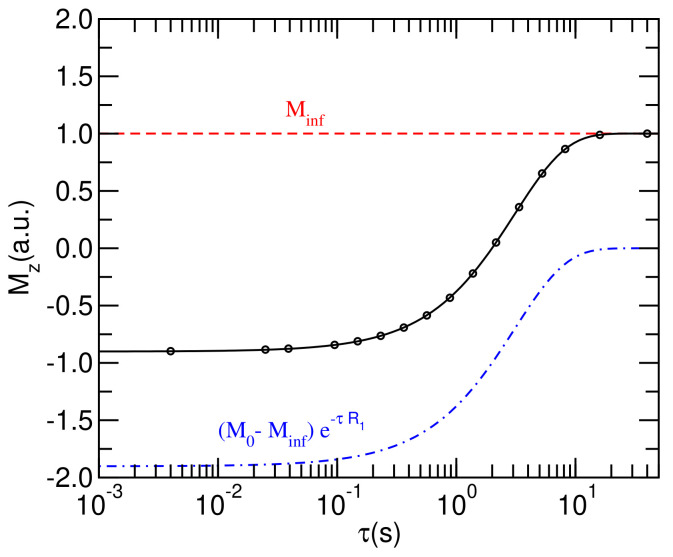
Sample curve showing the magnetization recovery for each inversion recovery delay, τ.

Generally, the results from relaxometry experiments are obtained by integrating over the entire ${}^{1}$H NMR spectrum and fitting the varying amplitudes, proportional to the magnetization along the fixed external magnetic field, to Equation (2). In Figure 1, the model fitting results following spectral integration are exemplified.

(2) $$M_{z}=M_{\infty }+\left(M_{0}-M_{\infty }\right)e^{-\tau R_{1}}$$

In the case of the present work, the high resolution spectrum, obtained at a 7T external magnetic field, was divided into the minimum number of Lorentzian components for which it was possible to determine the longitudinal relaxation rate and the fraction of the population corresponding to each contribution. The obtained results are presented in Appendix A.1.

**Figure A1:**
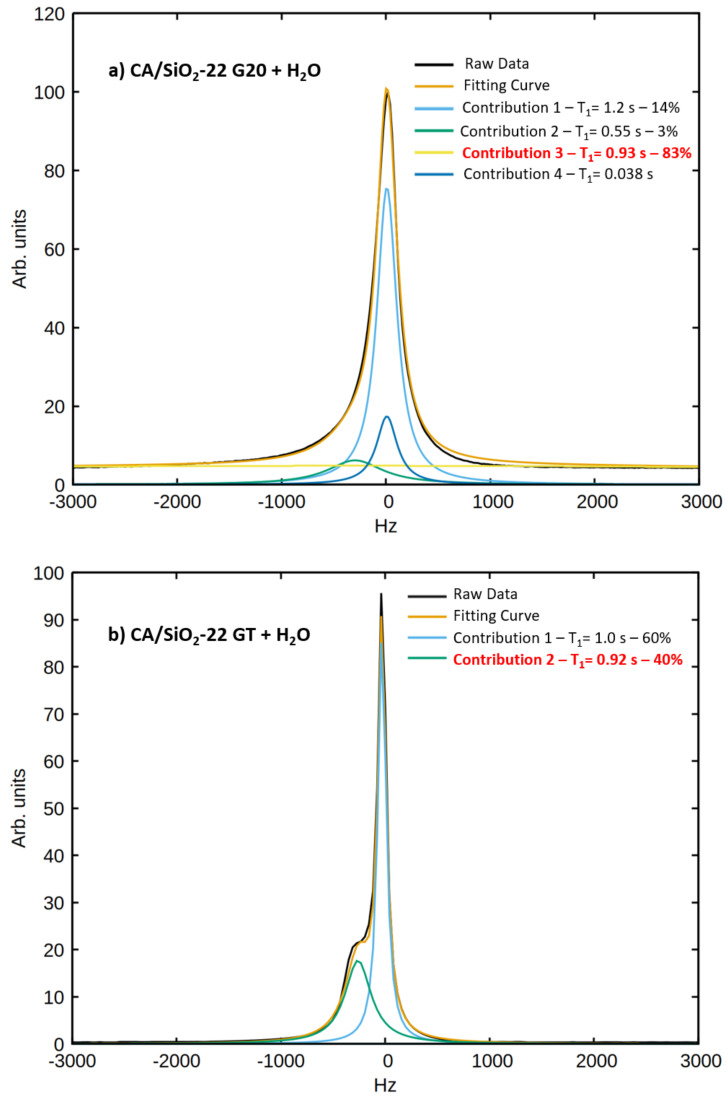
^1^H NMR spectroscopy analysis of the inversion recovery experimental spectra for membranes CA/SiO${}_{2}$-22 G20 (**a**) and GT (**b**).

**Figure A3:**
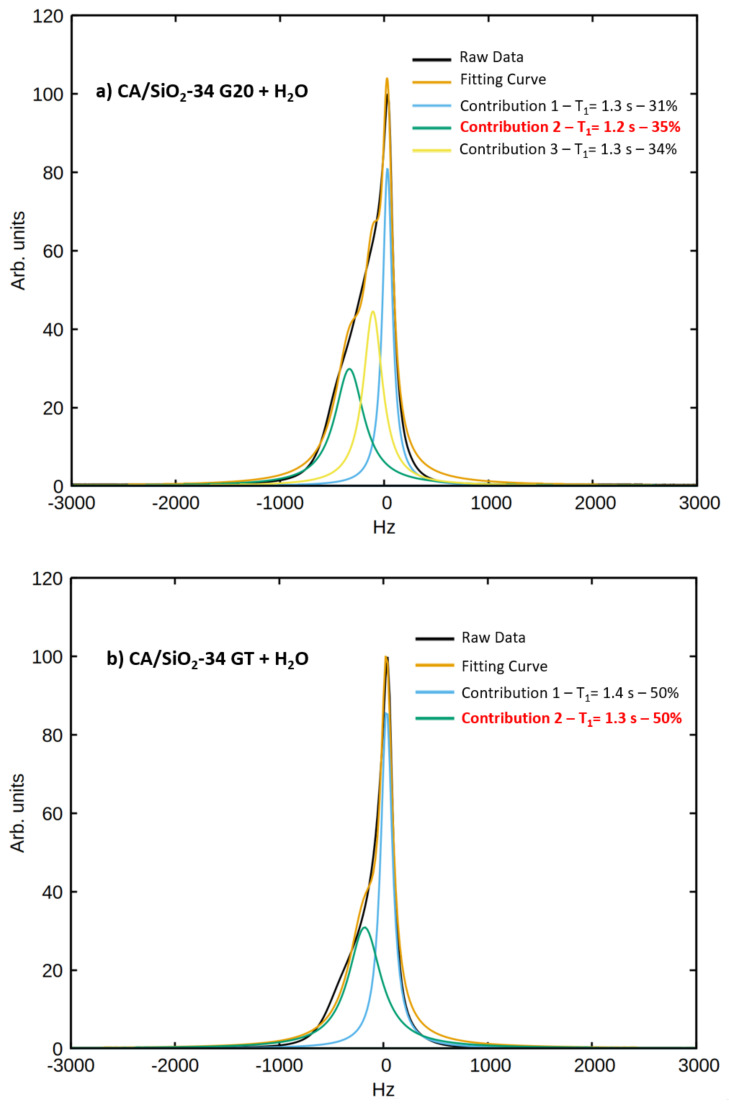
${}^{1}$H NMR spectroscopy analysis of the inversion recovery experimental spectra for membranes CA/SiO${}_{2}$-34 G20 (**a**) and GT (**b**).

For the majority of the studied systems, two components are observed. In these cases, the fraction of more confined water molecules, *q*, relates with the shortest relaxation time, T${}_{1}$ = R${}_{1}^{-1}$, which is highlighted in red in Figure A1–Figure A3. For samples CA/SiO${}_{2}$-22 G20 and CA/SiO${}_{2}$-34 G20, four and three contributions were, respectively, detected. In the case of sample CA/SiO${}_{2}$-22 G20, one of the contributions was immediately disregarded in view of the extremely small T${}_{1}$ (0.038 s), which would make it undetectable at lower frequencies. The other contribution having the shortest longitudinal relaxation time represented a very small percentage of the signal (3%), and it was, therefore, also not considered. For the CA/SiO${}_{2}$-34 G20 system, we simply considered the contribution with the shortest relaxation time to be that of water molecules in a more confined environment. The list of more confined water population fraction is presented in Table 3.

As it can be immediately concluded from the results presented in Table 3, the CA/SiO${}_{2}$-22 G20 membrane is dramatically different from CA/SiO${}_{2}$-30 G20 and CA/SiO${}_{2}$-34 G20 systems in terms of more confined population fraction or, in other words, the surface-to-volume ratio is much larger for CA/SiO${}_{2}$-22 G20. This result is consistent with the smaller pores observed for the CA/SiO${}_{2}$-22 membranes and the consequent lower hydraulic permeability of this system (see Table 2). The post-treatment with triton X-100 (GT) appears to have uniformized the confined population ratio for the three membrane compositions.

### 3.3. ^1^H NMR Diffusometry

**Figure 2:**
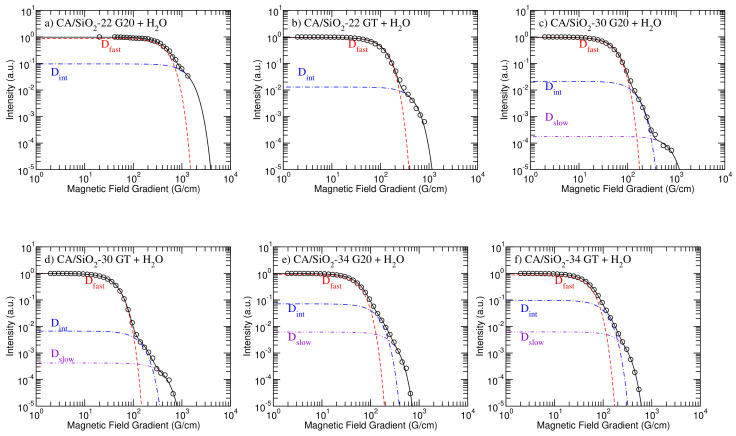
Diffusometry model fitting results obtained for the G20 and GT versions of membranes CA/SiO${}_{2}$-22—(**a**,**b**); CA/SiO${}_{2}$-30—(**c**,**d**); and CA/SiO${}_{2}$-34—(**e**,**f**)—membranes at 22 °C. The dashed-red line represents the fast diffusion contribution, the dot-dashed-blue line represents the intermediate diffusion contribution and the dot-dot-dashed-violet line represents the slow diffusion contribution.

Figure 2 shows the model fitting analyses made for each of the studied hydrated membranes, and Table 4 presents the obtained diffusion coefficients. The model fitting to the diffusometry and relaxometry data was performed using the open access online platform at fitteia.org (accessed in 1 September 2022), *fitteia*^®^, which applies the non-linear least squares minimization method with a global minimum target provided by the powerful MINUIT numerical routine from the CERN library [29,30].

As it can be observed, all hydrated membranes present at least two diffusion coefficients that can be associated with the water molecules experiencing different degrees of confinement. For the CA/SiO${}_{2}$-30 (G20 and GT) and CA/SiO${}_{2}$-34 (G20 and GT) systems, three diffusion components were observed. In all these cases, the third residual component can only be observed in the logarithmic scale. In the case of CA/SiO${}_{2}$-22 (G20 and GT), the slowest component is probably not observable due to its smaller value, which may fall out of the measurable range for this technique.

From Table 4, it is possible to conclude that the CA/SiO${}_{2}$-22 systems present much smaller diffusion coefficients than the CA/SiO${}_{2}$-30 and CA/SiO${}_{2}$-34 systems, which is expected in view of their smaller pores. Membranes CA/SiO${}_{2}$-30 and CA/SiO${}_{2}$-34 seem to be harder to distinguish in terms of the diffusion coefficient, possibly because their higher permeability increases the relative amount of less confined water. The slower and intermediate diffusion coefficients, $D_{slow}$ and $D_{int}$, respectively, seem to be smaller for CA/SiO${}_{2}$-30 systems, which is consistent with the smaller pore sizes observed for these membranes [15]. However, the faster diffusion component is larger for membranes CA/SiO${}_{2}$-30 than for membranes CA/SiO${}_{2}$-34, which might be a consequence of a the pore size distribution in membranes CA/SiO${}_{2}$-30 varying across a broader range of characteristic lengths. The fact that previous SEM studies have shown a wide distribution of pore sizes in these membranes makes it difficult to compare the ${}^{1}$H NMR diffusometry results obtained for membranes CA/SiO${}_{2}$-30 and CA/SiO${}_{2}$-34 [15]. Nevertheless, the CA/SiO${}_{2}$-22 systems are markedly less permeable and lead to much smaller diffusion coefficients, rendering the comparison between this and the CA/SiO${}_{2}$-30 and CA/SiO${}_{2}$-34 systems meaningful.

### 3.4. ^1^H NMR Relaxometry

#### 3.4.1. Raw Data and Theoretical Models

**Figure 3:**
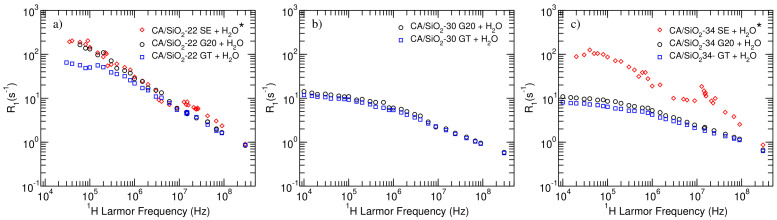
NMRD profiles obtained for the CA/SiO${}_{2}$-22—(**a**); CA/SiO${}_{2}$-30—(**b**); and CA/SiO${}_{2}$-34—(**c**)—membranes at 22 °C. (*) Data extracted from previous works by de Pinho et al. [16] related to membranes dried using the solvent exchange procedure.

In Figure 3, the ${}^{1}$H NMR relaxometry profiles obtained for the membranes studied in the present work are obtained. In order to enable a comparison between the profiles of membranes that were subject to a different drying process, the results previously obtained by de Pinho et al. [16] for membranes dried using the solvent exchange procedure were also added to the figure.

As it can be immediately concluded from the observation of the longitudinal relaxation profiles displayed in Figure 3, for the systems studied in the present work (black circles—G20—and blue squares—GT), the CA/SiO${}_{2}$-30 and CA/SiO${}_{2}$-34 membranes present rather similar relaxometry profiles, while CA/SiO${}_{2}$-22 membranes presented significantly different results.

It is also possible to see that post-treatment with triton X-100 leads to very small differences for CA/SiO${}_{2}$-30 and CA/SiO${}_{2}$-34 membranes, while it produces a significant $R_{1}$ decrease across the lower frequency range in the case of membranes CA/SiO${}_{2}$-22.

Furthermore, comparing the results obtained in the present work with those related to the solvent-exchange—(SE) dried membranes (red diamonds)—it is possible to observe a significant difference for the CA/SiO${}_{2}$-34 membranes while the CA/SiO${}_{2}$-22 systems seem almost insensitive to the drying process. This result may be explained by the fact that membranes that are more permeable, such as CA/SiO${}_{2}$-34, are bound to be more impacted by the drying process than systems with smaller pores. In fact, the hydraulic permeabilities found for these systems, reported in previous studies, also show that the permeability of the CA/SiO${}_{2}$-22 membrane is almost unaffected by post-treatment drying processes, while permeabilities obtained for the CA/SiO${}_{2}$-30 and CA/SiO${}_{2}$-34 systems vary over a wider range of values, especially when comparing the SE drying process to the G20 and GT post-treatments [15].

The curves presented in Figure 3 representing the longitudinal relaxation rate, $R_{1}$, obtained at different magnetic fields (or ${}^{1}$H Larmor frequencies) and called NMR dispersion (NMRD) curves encode information on the molecular dynamics of the systems under analysis. In the present work, it was considered that the water entrapped in the membranes’ pores may relax as a result of rotational and translational diffusions and rotations mediated by translational displacements, which are motivated by the interactions of water molecules with the porous matrix. Furthermore, assuming that these mechanisms are effective at different time scales and, thus, independent of one another, the total relaxation rate may be written as the sum of the individual rates (Equation (3)):

(3) $$R_{1}=R_{1}^{Rot}+\left(1-q\right)R_{1}^{SD}+qR_{1}^{RMTD},$$

where *q* is the fraction of water molecules interacting with the pore walls, which was determined with the analysis of the spectral components of the signals obtained at a ${}^{1}$H Larmor frequency of 300 MHz:

- Rotational diffusion (Rot):
  The model by Bloembergen, Purcell and Pound, better known as the BPP model, was applied in order to describe rotations of water molecules in the membranes [31,32]. The contribution of this mechanism to the NMR dispersion curves of water ${}^{1}$H spins is given by Equation (4).
  (4) $$R_{1}^{Rot}=A_{Rot}\left[\frac{\tau_{Rot}}{1+\omega^{2}\tau_{Rot}^{2}}+\frac{4\tau_{Rot}}{1+4\omega^{2}\tau_{Rot}^{2}}\right].$$
  The prefactor $A_{Rot}$ depends on the effective intramolecular distance between ${}^{1}$H nuclear spins, $r_{eff}$ (1.58 Å in the case of the water molecule), via Expression (5), which can easily be calculated for the water molecule:
  (5) $$A_{Rot}=\frac{3}{10}{\left(\frac{\mu_{0}}{4\pi }\right)}^{2}\gamma_{I}^{4}\hbar^{2}\frac{1}{r_{eff}^{6}},$$
  with $\mu_{0}$ denoting the vacuum magnetic permeability ($4\pi \times 10^{-7}$ H/m), $\gamma_{I}$ denoting the magnetic ratio of the nucleus with spin *I* and ℏ=h/(2π) denoting the reduced Planck constant ($1.0545718\times 10^{-34}$ m${}^{2}$Kg/s). Given that $A_{rot}$ can be estimated and fixed, the only parameter in Equation (4) that needs to be determined via the model-fitting analysis is the rotational correlation time, $\tau_{Rot}$.
- Translational Diffusion (SD):
  Self-diffusion of water molecules may be accounted for using the Torrey model [33,34]. Torrey assumed that molecules have equal probabilities of jumping in any direction from an initial state into another, reaching a random jump-like solution. The associated longitudinal relaxation rate frequency dependence is described by Equation (6).
  (6) $$R_{1}^{SD}=\frac{3}{2}{\left(\frac{\mu_{0}}{4\pi }\right)}^{2}\gamma_{{}^{I}}^{4}\hbar^{2}I\left(I+1\right)\left[j^{\left(1\right)}\left(\omega ,\tau_{D},d,r,n\right)+j^{\left(2\right)}\left(2\omega ,\tau_{D},d,r,n\right)\right].$$
  Parameter *n* is the ${}^{1}$H spin density, and *d* is the average intermolecular interspin distance. $\tau_{D}$, the translational diffusion correlation time, $<r^{2}>$, the mean square jump distance, and the diffusion coefficient, *D*, are related by the following equation.
  (7) $$<r^{2}>=6\tau_{D}D.$$
  The functions $j^{\left(i\right)}(\omega ,\tau_{D},d,r$, and n) are the spectral density functions described in references [33,34].
- Rotations mediated by translational displacements (RMTD):
  The water motion in the confined system gives rise to a relaxation mechanism associated with rotations mediated by translational displacements. This model describes the movement of water molecules near the pores’ walls and, therefore, is related to the interaction of those molecules with the membranes’ surfaces. The contribution of this model to the longitudinal relaxation rate is given by [35,36] the following:
  (8) $$\begin{array}{cc} R_{1}^{RMTD} & =\frac{A_{RMTD}}{\nu^{p}}G\left(\nu ,\nu_{max},\nu_{min}\right)\\ \; & =A_{RMTD}\left[\frac{f\left(\frac{\nu_{max}}{\nu }\right)-f\left(\frac{\nu_{min}}{\nu }\right)}{\nu^{p}}+4\frac{f\left(\frac{\nu_{max}}{2\nu }\right)-f\left(\frac{\nu_{max}}{2\nu }\right)}{2\nu^{p}}\right], \end{array}$$
  where
  (9) $$f\left(x\right)=\frac{1}{\pi }\left[arctan\left(\sqrt{2x}+1\right)+arctan\left(\sqrt{2x}-1\right)-arctan\left(\frac{\sqrt{2x}}{x+1}\right)\right]$$
  This contribution exhibits one high cut-off frequency, $\nu_{max}$, and one low cut-off frequency, $\nu_{max}$, which are, respectively, associated with the largest and smallest possible translational relaxation modes and, therefore, to the smallest and largest possible average displacements, respectively: $\nu_{max}^{-1}=l_{min}^{2}\pi /2D$ and $\nu_{min}^{-1}=l_{max}^{2}\pi /2D$, where *D* is the diffusion coefficient and *l* is the average displacement. Exponent *p* can vary between 0.5 and 1, where *p* = 0.5 corresponds to a situation where there is an isotropic distribution of coupled rotations and self-diffusion motions along the pore/channel’s surfaces, while for *p* = 1, there is a preferential orientation of the rotations/translations relaxation modes along the constraining surfaces. The parameter $A_{RMTD}$ is inversely proportional to the square root of the diffusion coefficient and to the range of wave numbers related to the motional modes induced by the surface, Δq. This parameter is proportional to the square of the fraction of molecules interacting with the surface and to the square of the order parameter, representing the long time limit residual correlation of restricted tumbling.

#### 3.4.2. Model Fitting

**Figure 4:**
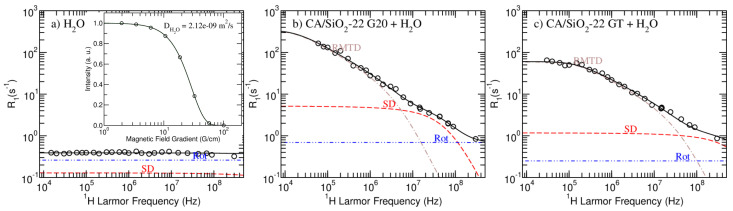
NMRD profiles and model fitting results obtained for the pure water—(**a**); CA/SiO${}_{2}$-22 G20—(**b**); and CA/SiO${}_{2}$-22 GT—(**c**)—membranes. The dot-dashed-brown lines represents the RMTD contribution, the dashed-red-line represents the self-diffusion contributions and the dot-dot-dashed-blue line represents the rotations/reorientations contribution to the longitudinal relaxation rate profiles.

**Figure 5:**
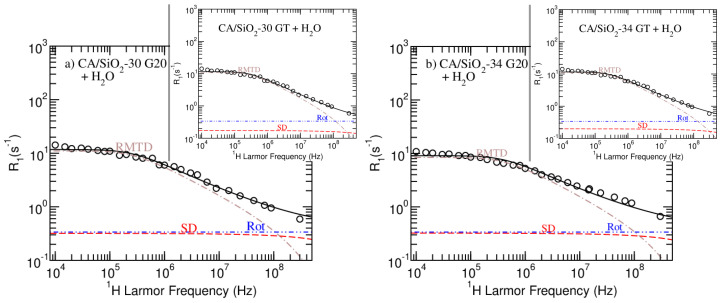
NMRD profiles and model fitting results obtained for systems CA/SiO${}_{2}$-30 G20 and GT—(**a**); and CA/SiO${}_{2}$-34 G20 and GT—(**b**). The dot-dashed-brown lines represents the RMTD contribution, the dashed-red-line represents the self-diffusion contributions and the dot-dot-dashed-blue line represents the rotations/reorientations contribution to the longitudinal relaxation rate profiles.

In Figure 4 and Figure 5, the model fitting results produced by the *Master* module of the online platform *fitteia*^®^ [37] are presented. Figure 4 show the results obtained for pure water and the CA/SiO${}_{2}$-22 G20 and GT-hydrated membranes. The model fitting analysis of CA/SiO${}_{2}$-30 and CA/SiO${}_{2}$-34 systems is presented in Figure 5. The model fitting parameters resulting from the NMRD curves analysis are summarized in Table 5.

The model proposed in this work and the combination of ${}^{1}$H NMR relaxometry and diffusometry experimental techniques allowed for a consistent analysis of all the studied hydrated membranes, as it can be concluded by the good quality of the fits.

In Figure 4, it is possible to observe the striking NMRD profile difference when comparing pure water with confined water. Water molecules entrapped in the matrix have the additional RMTD relaxation pathway, which significantly increases the longitudinal relaxation rate. Moreover, confined water presented diffusion coefficients that are up to three orders of magnitude smaller than that measured for free water (see inserted image in Figure 4a and Table 5).

The parameter *q* was fixed to the value obtained from the analysis of the spectral components. $D_{SD}$ and $D_{RMTD}$ were set equal to the value of $D_{fast}$ and $D_{int}$ obtained from the diffusometry analysis and presented in Table 4, respectively. $D_{SD}$ corresponds to a less confined fraction of water that does not interact directly with the matrix, while $D_{RMTD}$ corresponds to a more confined fraction of water that relaxes as a result of interactions with the surface.

Despite the apparent similarities between the relaxometry profiles obtained for the G20 and GT versions of membranes CA/SiO${}_{2}$-30 and CA/SiO${}_{2}$-34, the model-fitting analysis evidences a decrease in the self-diffusion relaxation rate contribution for the membranes that were post-treated with triton X-100 (compare the dashed red line of the sub figures with that of the respective inserted image in Figure 5). This contribution decrease is more significant for the CA/SiO${}_{2}$-22 membranes, as observed in Figure 4. This observation is consistent with triton X-100 increasing the hydrophobicity of the cellulose acetate matrix, making the water less bound to it and leading to higher diffusion coefficients (see $D_{SD}$ and $D_{RMTD}$ in Table 5 or, respectively, $D_{fast}$ and $D_{int}$ in Table 4). The fact that this increase is more significant for the CA/SiO${}_{2}$-22 hydrated membranes may be explained by the fact that smaller pore sizes relate to a larger ratio of water/surface interactions.Furthermore, the increased hydrophobicity suggested from this model-fitting analysis might explain the uniformization of the bound water fraction, *q*, found for the GT porous membranes (see Table 3).

This analysis enabled the estimation of the characteristic pore size given by the parameter $l_{max}$, that, on average, induces more effective ${}^{1}$H NMR relaxation through rotations mediated by translational displacements. As it can be observed, the additional treatment with triton X-100 does not significantly affect this dimension, except in the case of CA/SiO${}_{2}$-34 systems. Combining the previously described increased hydrophobicity with the fact that this membrane is the most permeable, it is possible that the signal from more bound water molecules is masked by the signal of unbound water, leading to an apparently larger characteristic dimension.

Regarding the fact that $A_{RMTD}$ is inversely proportional to the square root of the diffusion coefficient, the values obtained for this parameter seem to be consistent for all the samples and further support the increased hydrophobicity conferred upon treating the matrix with triton X-100 (GT).

Parameter *p* shows that there is an isotropic distribution of coupled rotations and self-diffusion motions along the matrix’ pores for all systems, except for the CA/SiO${}_{2}$-22 pair, where some degree of anisotropy is detected. The fact that CA/SiO${}_{2}$-22 membranes have smaller pores is expected to increase the degree of confinement, thus evidencing water-ordering induced by the surface.

## 4. Conclusions

In this study, ${}^{1}$H NMR spectroscopy, diffusometry and relaxometry were successfully combined in order to consistently analyze three pairs of hydrated ultrafiltration/nanofiltration asymmetric cellulose acetate–silica membranes. Each CA/SiO${}_{2}$-22, CA/SiO${}_{2}$-30 and CA/SiO${}_{2}$-34 pair of membranes was composed of one membrane in which the post-treatment involved an aqueous solution of glycerol with 4 vol.% of triton X-100 (GT) and another where triton was not involved in the post- treatment (G20).

The results seem to be consistent with the post-treatment with triton X-100 rendering the matrix surfaces more hydrophobic and increasing the self-diffusion coefficients obtained for water molecules in different confinement environments. This impact is more significant when the characteristic pore sizes are smaller given the increased probability of water/matrix interactions.

Comparing the results obtained in the present work with those related to membranes dried using the solvent-exchange procedure, presented in previous studies, it becomes clear that the drying process has a much less pronounced impact on the cellulose acetate–silica matrix when the pores are characterized by smaller dimensions.

The surface-bound water population variation observed between the CA/SiO${}_{2}$-22 G20 and the two analogous membrane systems is in line not only with the diffusion coefficients obtained in the present work but also with the hydraulic permeabilities reported in previous studies.

On the whole, this work evidences the advantage of combining complementary experimental techniques with a relatively simple relaxation model to study and differentiate between ultrafiltration/nanofiltration porous media and track their sensitivity to different post-treatments/drying processes.

## Figures and Tables

**Table 1:** Asymmetric CA/SiO_2_ hybrid membranes film casting solutions and casting conditions.

Casting Solution Composition (wt.%)			
Membrane	CA/SiO_2_-22	CA/SiO_2_-30	CA/SiO_2_-34
CA	16.4	16.4	16.4
Formamide	21.3	29.0	32.9
Acetone	58.8	51.1	47.2
TEOS (SiO_2_ precursor)	3.0	3.0	3.0
H_2_O	0.5	0.5	0.5
HNO_3_	4 drops (pH ≈ 2)	4 drops (pH ≈ 2)	4 drops (pH ≈ 2)
**Casting Conditions**			
Temperature of casting solution (°C)		20–25	
Temperature of casting atmosphere (°C)		20–25	
Relative humidity of casting atmosphere (%)		40–50	
Solvent evaporation time (min)		0.5	
Gelation medium		Ice-cold deionised water (2 h)	

**Table 2:** Characteristics of the asymmetric CA/SiO${}_{2}$ hybrid membranes [15].

Membrane		Hydraulic Permeability,	Molecular Weight Cut-Off,
		L${}_{p}$ (kg h${}^{-1}$ m${}^{-2}$ bar${}^{-1}$)	MWCO (kDa)
CA/SiO${}_{2}$-22	G20	3.5 ± 0.2	4
	GT	2.2 ± 0.2	3
CA/SiO${}_{2}$-30	G20	38 ± 2	14
	GT	40 ± 3	29
CA/SiO${}_{2}$-34	G20	81 ± 4	35
	GT	62 ± 4	21

**Table 3:** Considered fraction of more confined water molecules to apply in the ${}^{1}$H NMR relaxometry analysis.

	CA/SiO${}_{2}$-22		CA/SiO${}_{2}$-30		CA/SiO${}_{2}$-34	
	G20	GT	G20	GT	G20	GT
More confined population ratio, *q*	0.83	0.40	0.24	0.40	0.35	0.50

**Table 4:** Diffusion coefficients obtained from the PGSE ${}^{1}$H NMR experiments performed at 25 °C. The model fitting was performed considering by an uncertainty equal to 5% of the signal intensity for each point.

Membrane		$D_{fast}$ ( $10^{-10}$ $m^{2}$ /s)	$D_{int}$ ( $10^{-11}$ $m^{2}$ /s)	$D_{slow}$($10^{-11}$ $m^{2}$/s)
CA/SiO${}_{2}$-22	G20	0.09	0.11	–
	GT	1.40	0.97	
CA/SiO${}_{2}$-30	G20	6.50	9.50	0.38
	GT	9.50	9.60	1.10
CA/SiO${}_{2}$-34	G20	5.50	10.0	2.20
	GT	6.90	16.0	3.10

**Table 5:** Parameters obtained from the NMRD model fitting analysis made on the studied membranes. The model fitting was performed considering an uncertainty of the relaxation rate equal to 10% of its value. The ${}^{1}$H spin density, *n*, needed for the Torrey model for translational self-diffusion was fixed to the calculated value of 6.69 × 10${}^{28}$${}^{1}$H nuclear spins per cubic meter. Parameters $D_{SD}$ and $D_{RMTD}$ were fixed to the $D_{fast}$ and $D_{int}$ diffusion coefficients presented in Table 4, respectively. In the case of pure water, the diffusion coefficient was fixed to that presented in Figure 4. The fraction *q*, representing the more confined water, was also not a free parameters, and its value was set to that presented in Table 3 for each studied hydrated membrane. Parameter $A_{rot}$ was also calculated and fixed as explained in the rotations model section.

Parameters	${\text{CA/SiO}}_{2}$ -22		${\text{CA/SiO}}_{2}$ -30		${\text{CA/SiO}}_{2}$ -34		$H_{2}O$
	G20	GT	G20	GT	G20	GT	
$A_{Rot}(10^{10}$$s^{-2}$)	1.08		1.08		1.08		1.08
$\tau_{Rot}(10^{-12}$ s)	13	5	6		6		5
$D_{SD}(10^{-10}$$m^{2}$/s)	0.09	1.40	6.50	9.50	5.50	6.90	21
*r*(Å)	3.0		3.0		3.0		3.0
*d*(Å)	2.7		2.7		2.7		2.7
*q*	0.83	0.40	0.24	0.40	0.35	0.50	–
$D_{RMTD}(10^{-11}$$m^{2}$/s)	0.11	0.97	9.50	9.60	10.0	16.0	–
$A_{RMTD}(10^{3}$$s^{-(1+p)}$)	27	18	5.8	3.7	3.8	2.9	–
$l_{max}(10^{-9}$ m)	5.3	5.3	13	12	10	16	–
*p*	0.56	0.51	0.50		0.50		–

**Figure A2:**
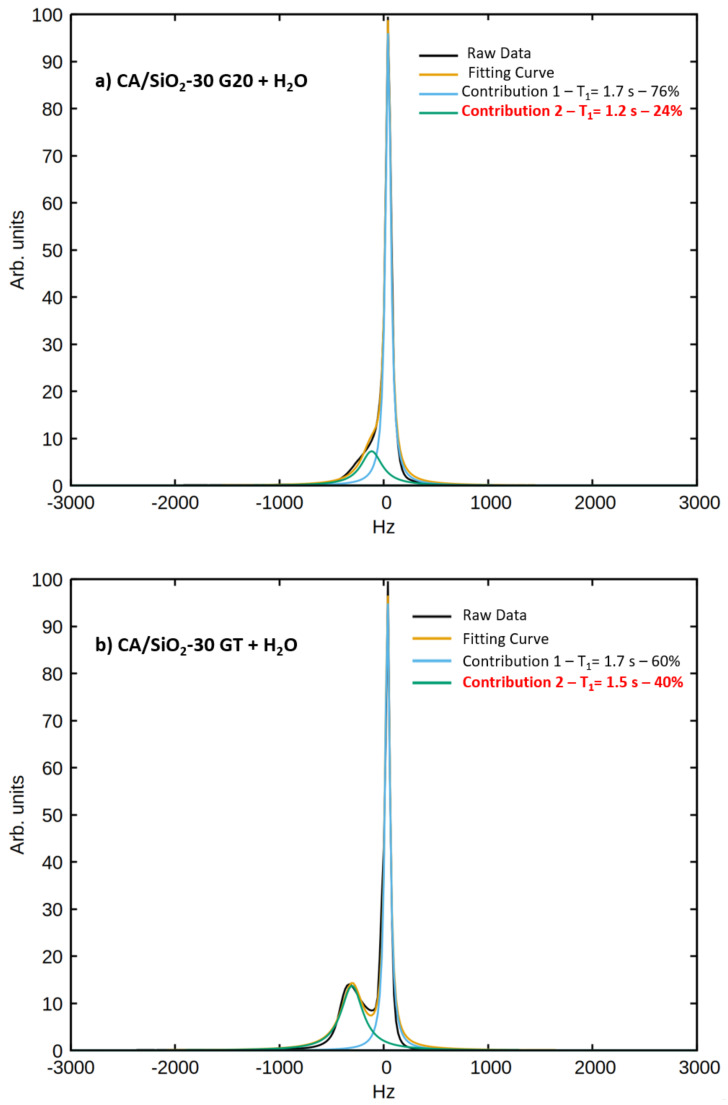
${}^{1}$H NMR spectroscopy analysis of the inversion recovery experimental spectra for membranes CA/SiO${}_{2}$-30 G20 (**a**) and GT (**b**).

## Data Availability

Not applicable.

## Abbreviations

The following abbreviations are used in this manuscript:

NMR	Nuclear magnetic resonance;
^1^H	Proton, hydrogen-1;
PGSE	Pulse Gradient Stimulated Echo;
Rot	Rotations;
SD	Self-diffusion;
RMTD	Rotations Mediated by Translational Displacements;
CA	Cellulose acetate;
NF	Nanofiltration;
UF	Ultrafiltration;
CA/SiO_2_	Cellulose acetate/silica;
G20	Surfactant conditioning with an aq. sol. of glycerol 20 vol.%;
GT	Surfactant conditioning with an aq. sol. of glycerol 20 vol.% and triton x-100 4 vol.%.
